# Identification and validation of novel prognostic signatures based on m5C methylation patterns and tumor EMT profiles in head and neck squamous cell carcinoma

**DOI:** 10.1038/s41598-023-45976-6

**Published:** 2023-10-31

**Authors:** Guanghao Zhu, Wei Wang, Hui Yao, Haopu Li, Caiyun Zhang, Yindi Meng, Jingjie Wang, Minhui Zhu, Hongliang Zheng

**Affiliations:** Department of Otolaryngology Head and Neck Surgery, Shanghai Changhai Hospital, The Second Military Medical University, Shanghai, China

**Keywords:** Head and neck cancer, Metastasis, Tumour biomarkers

## Abstract

The role of 5-methylcytosine (m5C) in tumor initiation and progression has been increasingly recognized. However, the precise association between the regulation of m5C and the progression, metastasis, and prognosis of head and neck squamous cell carcinoma (HNSCC) has not yet been fully explored. Data from 545 HNSCC patients obtained from The Cancer Genome Atlas (TCGA) database were analyzed. Unsupervised cluster analysis was conducted using the expression levels of m5C regulatory genes. Additionally, gene set variation analysis (GSVA), single-sample gene set enrichment analysis (ssGSEA), and Cox regression analysis were utilized. Quantitative reverse transcription polymerase chain reaction (RT-qPCR), colony formation assay, transwell experiments and western blots were performed in the HNSCC cell line UM-SCC-17B to assess the expression and functional role of one of the novel signatures, CNFN. Significant expression differences were found in m5C regulatory genes between tumor and normal tissues in HNSCC. Two distinct m5C modification patterns, characterized by substantial prognostic differences, were identified. Cluster-2, which exhibited a strong association with epithelial-mesenchymal transition (EMT), was found to be associated with a poorer prognosis. Based on the m5C clusters and EMT status, differentially expressed genes (DEGs) were identified. Using DEGs, an 8-gene signature (CAMK2N1, WNT7A, F2RL1, AREG, DEFB1, CNFN, TGFBI, and CAV1) was established to develop a prognostic model. The performance of this signature was validated in both the training and external validation datasets, demonstrating its promising efficacy. Furthermore, additional investigations using RT-qPCR on clinical specimens and experimental assays in cell lines provided compelling evidence suggesting that CNFN, one of the genes in the signature, could play a role in HNSCC progression and metastasis through the EMT pathway. This study highlighted the role of m5C in HNSCC progression and metastasis. The relationship between m5C and EMT has been elucidated for the first time. A robust prognostic model was developed for accurately predicting HNSCC patients’ survival outcomes. Potential molecular mechanisms underlying these associations have been illuminated through this research.

## Introduction

Head and neck squamous cell carcinoma (HNSCC) is the most common malignant tumor in the head and neck region, originating from the mucosal epithelium of the oral cavity, pharynx, and larynx. Globally, HNSCC accounts for approximately 900,000 new cases and 450,000 deaths annually, with China alone contributing around 80,000 new cases and 45,000 deaths^1,2^. HNSCC is characterized by insidious onset, high invasiveness, and high recurrence rates, significantly impacting patients’ quality of life. Research has shown that HNSCC patients have twice the risk of suicide compared with patients with other types of cancer^3^. There is currently no effective screening strategy for HNSCC. The majority of patients are diagnosed at an advanced stage of HNSCC, without any significant clinical history prior to diagnosis. This contributes to the generally poor prognosis of HNSCC patients^4^. Therefore, there is an urgent need for a predictive marker to aid in the diagnosis of HNSCC, predict its prognosis, and assist in subsequent treatment decisions.

RNA methylation is a common epigenetic regulatory mechanism in mammals, and it has been proven to play a noticeable role in modulating gene expression. To date, more than 170 RNA modification patterns have been identified, with methylation accounting for over half of the known modifications^5,6^. The main types of RNA methylation include N6-methyl-adenosine (m6A), 5-methylcytosine (m5C), N1-methyladenosine (m1A), N7-methylguanosine (m7G), etc^7,8^. Among them, m5C is a widely studied RNA modification that can be found in tRNA, rRNA, mRNA, and non-coding RNA^9^. The regulation of m5C is a dynamic process controlled by three main regulators called 'writers' for adding specific modifications, 'readers' for recognizing and binding modified nucleotides, and 'erasers' for removing specific modifications^10^. RNA m5C methyltransferases primarily include NOL1/NOP2/sun (NSUN) methyltransferases and the DNA methyltransferase-like protein DNMT2. They catalyze m5C modification on RNA, which then interacts with specific recognition proteins to exert specific biological functions^11^. It has been demonstrated that m5C methylation is associated with the progression, metastasis, and drug resistance of various types of cancer. For instance, m5C methylation of SUMO-2/3 has shown to contribute to the progression of gastric cancer, and high levels of m5C methylation could lead to mRNA overexpression in diverse types of cancer^12,13^. Furthermore, a recent study has demonstrated that NSUN3-mediated mitochondrial RNA methylation could play a crucial role in cancer metastasis^14^. To summarize, the function of RNA m5C methylation is noticeable that is implicated in the progression and metastasis of cancer.

Epithelial-mesenchymal transition (EMT) represents another significant mechanism contributing to tumor metastasis and unfavorable prognosis. EMT is a process by which epithelial cells acquire mesenchymal characteristics. In cancer, EMT is associated with tumor initiation, invasion, metastasis, and resistance to therapy^15^. The EMT process includes loss of adhesion, cytoskeletal remodeling, evasion of apoptosis and immune surveillance, upregulation of metalloproteinases, neovascularization, acquisition of stem cell-like properties, and activation of the tumor microenvironment^16,17^. This change can make the cancer easier to invade surrounding tissues and spread to other areas of the body. In numerous types of tumors, the advancement of the disease is mainly associated with the activation of EMT. EMT provides tumor cells with the ability to initiate migration, which is a key step in metastasis. Notably, studies have shown that there is a significant upregulation of EMT-related genes in metastatic lesions of patients with breast cancer, ovarian cancer, and pancreatic cancer. This underscores the potential importance of EMT in promoting metastasis in these particular types of cancer^18–20^. Moreover, the involvement of EMT in the metastatic process of HNSCC has been assessed^21^. Studies have shown that downregulation of E-cadherin could transform low-invasive tumors into highly invasive ones, while overexpression of E-cadherin could significantly reduce tumor invasion and migration capabilities^22,23^. However, whether RNA m5C methylation modification is involved in the regulation of tumor EMT process has not yet been comprehensively investigated and requires further research.

In the present study, an analysis of HNSCC patients’ data from The Cancer Genome Atlas (TCGA) database was conducted based on the expression levels of m5C regulatory genes. Two distinct m5C modification patterns associated with different prognostic outcomes were identified. Additionally, it was found that these different m5C modification patterns were correlated with varying levels of EMT. According to the m5C modification and EMT levels, an 8-gene signature was developed to predict the prognosis of HNSCC and experiments were conducted to explore potential molecular mechanisms. This research may contribute to the diagnosis of HNSCC, deepen our understanding of the disease, and provide novel insights into potential therapeutic targets for improving patients’ prognosis.

## Materials and methods

### Patients and follow-up

Totally, 20 patients with HNSCC who were admitted to Changhai Hospital, Naval Military Medical University (China) from January 2017 to January 2022 were involved in this study.

### HNSCC data acquisition and preprocessing

The gene expression from TCGA-HNSC, measured as fragments per kilobase per million mapped reads (FPKM), were obtained from TCGA database (http://gdc.cancer.gov). The TCGA phenotype data of HNSC were obtained through Xena platform and are presented in “Supplementary Table 1”^24^. We removed samples with missing values and non-expressed genes exceeding 50% of the total number of sequenced samples, where non-expressed genes are genes with zero expression. After excluding samples with missing survival time and those with more than 50% of non-expressed genes, a total of 545 samples were included in the analysis. Among them, there were 501 tumor samples and 44 normal samples.

The GSE65858 dataset, which utilizes the sequencing platform GPL10558 Illumina HumanHT-12 V4.0 expression beadchip, was retrieved from the National Center for Biotechnology Information Gene Expression Omnibus (GEO) database (https://www.ncbi.nlm.nih.gov)^25^. After removing samples with missing survival time and those with zero survival time, a total of 270 HNSCC samples with available prognostic information were selected for further validation analysis.

### Mapping of m5C regulatory genes

A total of 16 m5C regulatory genes, including 11 writers (TRDMT1, NOP2, NSUN2, NSUN3, NSUN4, NSUN5, NSUN6, NSUN7, DNMT1, DNMT3A, DNMT3B), 3 errs (TET1, TET2, TET3), and 2 readers (YBX1, ALYREF), were summarized from the currently published studies^26–28^. Then, the differences in expression levels of m5C regulatory genes between HNSCC samples and normal samples were identified, followed by assessment of the mutation frequency of m5C regulatory genes using maftools R package^29^, and mapping of m5C regulatory genes mapping was carried out via RCircos R package^30^.

### Unsupervised cluster analysis

According to the above-described m5C regulatory genes, unsupervised cluster analysis was performed using ConsensusClusterPlus R package^31^. To obtain the best m5C cluster (K-value), the K-value ranged from 2 to 6. After that, the m5C score of each TCGA sample was evaluated using GSVA R package^32^, and the Wilcoxon test was then utilized to compare the differences in the distribution of m5C scores among different m5C clusters.

### Gene set enrichment analysis (GSEA)

The GSEA is a computational approach that evaluates whether a specific gene set exhibits statistically significant differences between two biological conditions. In this study, GSEA was employed to examine the enrichment of genome-wide significant hallmark gene sets (h.all.v7.4.symbols) among different m5C clusters. A cut-off threshold was set, considering only results with a P-value less than 0.05 and a normalized enrichment score (NES) greater than 1 as significant.

### Estimation of EMT score

In this study, the Msigdb database (http://www.gsea-msigdb.org/gsea/msigdb/index.jsp) was used to retrieve the relevant EMT pathway genes with "HALLMARK_EPITHELIAL_MESENCHYMAL_TRANSITION" as the keyword, and the relevant EMT pathway gene sets were retrieved. The EMT score of each head and neck squamous cell carcinoma patient was then estimated using the ssGSEA method in the R package "GSVA".

### Identification of differentially expressed genes (DEGs)

Differential gene expression analysis was carried out using the "limma" R package for different m5C clusters and EMT score groups. The differential expression threshold was set as follows: |fold-change (FC)|≥ 1.5 and adjusted P-value < 0.05. The "Venn" R package was utilized to extract the overlapping genes between these two sets as the gene set for subsequent analysis.

### Development of the risk prediction model

A risk prediction model was developed using the overlapping genes through a series of statistical analyses. First, univariate Cox regression analysis was performed to identify the potential predictors. Then, the least absolute shrinkage and selection operator (LASSO) regression analysis was employed to further refine the model by selecting the most informative genes. Finally, stepwise Cox regression analysis was conducted to establish the risk score based on the regression coefficients of each gene and their corresponding expression levels in the model. The risk score was formulated as follows:

$$Risk = \beta_{1} * Gene_{1} + \beta_{2} * Gene_{2} + ... + \beta_{r} * Gene_{r}$$

where β_1_, β_2_, …, β_r_ represent the regression coefficients of the genes (obtained from the stepwise Cox regression analysis), and Gene_1_, Gene_2_, …, Gene_r_ represent the expression levels of the corresponding genes.

### Cell culture

UM-SCC-17B cells were sourced from the University of Michigan and subjected to STR identification to confirm their authenticity. Additionally, the cells were tested for mycoplasma contamination to ensure their purity. The cells were cultured in a Dulbecco’s modified Eagle’s medium (DMEM) supplemented with 10% fetal bovine serum (FBS) and 1% penicillin/streptomycin (P/S). The cell culture was maintained at a temperature of 37 °C in a humidified atmosphere with 5% CO_2_ to provide optimal growth conditions.

### Cell transfection

The cells were transfected with small-interfering RNA (siRNA) against CNFN (siCNFN-308) and corresponding negative control siRNA (siCtrl). All siRNAs were synthesized by GenePharma Co., Ltd. (Shanghai, China). Transfection of cells was performed using jetPRIME transfection reagent (Polyplus Co., Ltd., Paris, French) once they reached an appropriate confluency.

### Quantitative reverse transcription polymerase chain reaction (RT-qPCR)

Total RNA from tissue and cells was isolated using TRIzol reagent (Thermo Fisher Scientific, Waltham, MA, USA) according to the manufacturer’s instructions. Subsequently, cDNA was synthesized using the Evo M-MLV RT kit with gDNA Clean for qPCR (Accurate Biology, China). The RT-qPCR was conducted on an ABI7500 instrument (Thermo Fisher Scientific) using the Universal Blue SYBR Green qPCR Master Mix kit (Servicebio Technology Co., Ltd., Wuhan, China). The expression levels of the target genes were normalized to glyceraldehyde 3-phosphate dehydrogenase (GAPDH), which served as an internal reference. The data were analyzed using the 2^−ΔΔCt^ method.

### Cell counting kit-8 (CCK-8) assay

In brief, 10^3 cells were seeded into each well of a 96-well plate and cultured for a specific period of time. For detection, 10 μL of CCK-8 reagent was added to each well, followed by a 4-h incubation. The cell viability was then assessed using a microplate reader (Thermo Fisher Scientific) at an absorbance of 450 nm.

### Colony formation assay

After cellular transfection, 1.2 × 10^3 cells were seeded into a six-well plate for culture. The culture medium was replaced every 3 days. After 2 weeks, the cells were fixed with 4% paraformaldehyde and stained with crystal violet. The number of colonies containing more than 50 cells was counted.

### Wound healing assay

After cellular transfection, UM-SCC-17B cells were transferred to a 96-well plate and cultured until reaching 90% confluence. A scratch of the same size was created using the AccuWound 96 Scratch Tool (Agilent Technologies Inc., Santa Clara, CA, USA), followed by washing with phosphate-buffered saline (PBS) and replacing with a serum-free culture medium. After 24 h, the scratch area was scanned using the ArrayScan HCA Reader (Thermo Fisher Scientific), and quantification was performed using the HCS system.

### Migration and invasion assays

For the migration and invasion assays, Transwell chambers were used for evaluation (Corning Inc., Corning, NY, USA). For the migration assay, 2 × 10^4 cells in 200 μL of serum-free culture medium were added to the upper chamber, while the lower chamber was filled with 10% FBS-containing medium. For the invasion assay, the Transwell chambers were pre-coated with a Matrigel (Corning Inc., Corning, NY, USA), and 2 × 10^5 cells were added into the upper chamber. After 24 h of incubation, the cells on the lower surface of the membrane were fixed with 4% paraformaldehyde and stained with crystal violet. The migrated cells were observed and counted under an optical microscope.

### Western blotting

The proteins were separated by sodium dodecyl-sulfate polyacrylamide gel electrophoresis (SDS-PAGE) and transferred onto polyvinylidene difluoride (PVDF) membranes (Millipore, Billerica, MA, USA). After blocking with 5% skim milk, the membranes were incubated with primary antibodies against ZO-1 (21773-1-AP), E-cadherin (20874-1-AP), N-cadherin (22018-1-AP), Vimentin (10366-1-AP), and SNAI2 (12129-1-AP) at 4 °C for 12 h. Subsequently, the membranes were incubated with secondary antibodies for 1 h, and the protein bands were visualized using an ECL detection reagent (Thermo Fisher Scientific, Waltham, MA, USA) and captured by imaging. GAPDH was used as an internal control.

### Statistical analysis

The Student's t-test was utilized for making comparison between two groups, while one-way analysis of variance (ANOVA) and Kruskal–Wallis tests were used for making comparison among more than two groups. Kaplan–Meier curves were used to plot survival curves for prognostic analysis, and the log-rank test was applied to identify significant differences. A two-sided P < 0.05 was considered statistically significant. Statistical analysis was carried out using R 4.2.1 and GraphPad Prism 9.4.1 software.

### Ethics approval and consent to participate

All patients were informed with the experimental protocol and they all singed informed consent and all research was performed in accordance with relevant guidelines/regulations. All procedures were approved by Changhai Hospital, Navy Military Medical University Ethics Committee.

## Results

### Landmarks of the m5C regulator genes in HNSCC

**Figure 1:**
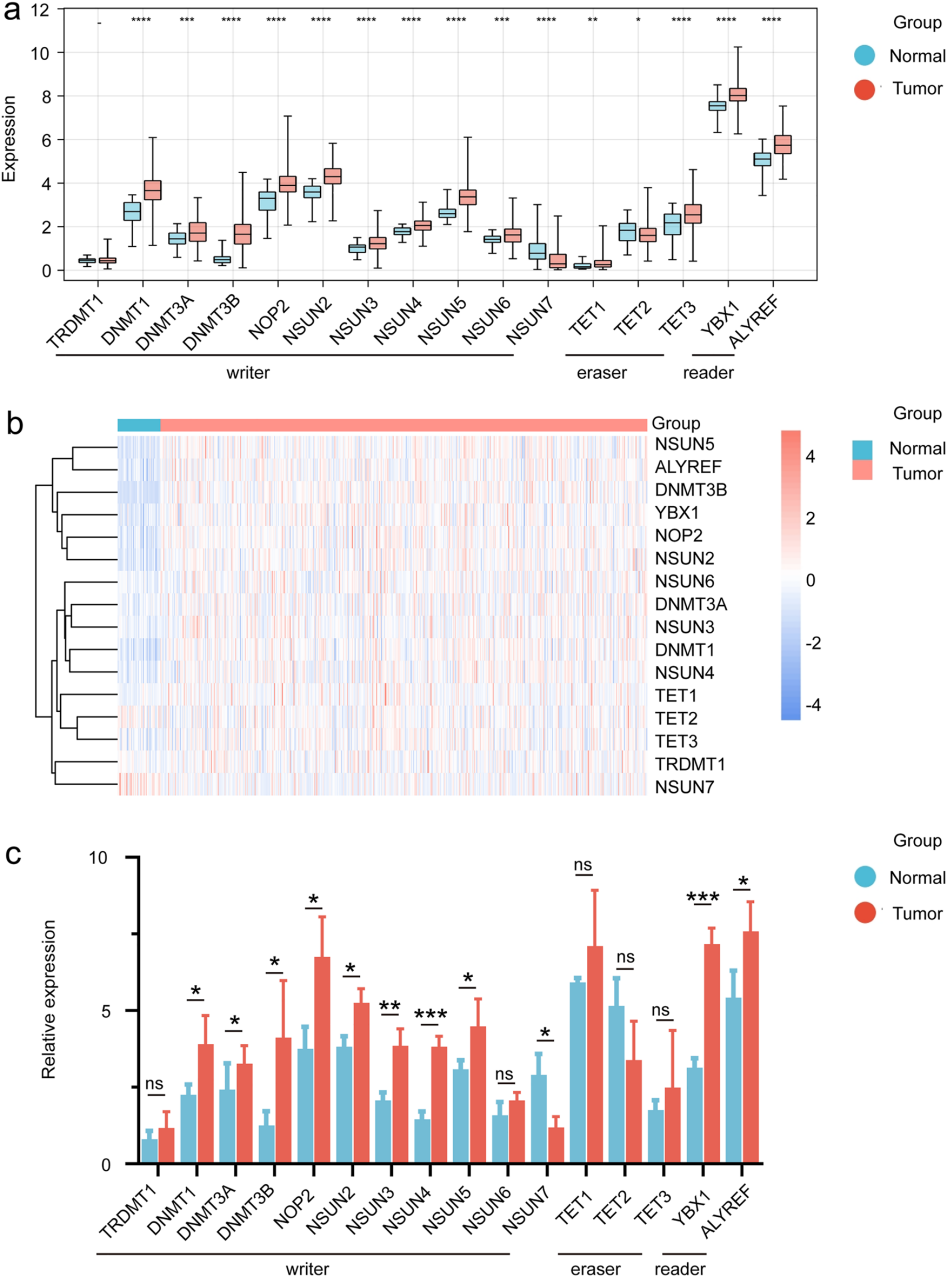
Expression mapping of m5C regulatory genes in HNSCC cohort and clinical samples. (**a**) Expression levels of the m5C regulatory genes in HNSCC and normal tissues; (**b**) Heatmap showing the expression levels of m5C regulatory genes in HNSCC and normal tissues. The heatmaps was constructed via the R package ggplot2; (**c**) Relative expression of 11 writers, 3 erasers and 2 readers of m5C genes based on RT-qPCR in clinical samples of tumor vs normal tissue.

Totally, 16 m5C regulatory genes, including 11 writers, 3 erasers, and 2 readers were identified. Firstly, the expression levels of m5C regulatory genes in HNSCC samples were measured. Figure 1a,b shows the expression of m5C regulatory genes in HNSCC. In all 11 writers, the expression of TRDMT1 was low in both tumor and normal tissues, and its expression was not significantly different in tumor vs. normal tissues. NOP2, NSUN2, NSUN3, NSUN4, NSUN5, NSUN6, DNMT1, DNMT3A, DNMT3B were significantly higher in tumors than in normal tissues, while NSUN7 was highly expressed in normal tissues. In erasers, TET1 and TET3 were highly expressed in tumor tissues, while TET2 was down-regulated in tumor tissues. Both readers were upregulated in tumors. RT-qPCR was used to detect the expression of these m5C regulators in our patients, and the information of these patients is presented in Table 1. These m5C regulators had similar expression patterns in clinical samples as in samples from TCGA. However, NSUN6, TET1, TET2, and TET3 did not have significant differences in tumor vs. normal tissues (Fig. 1c). In addition, the mutational status of m5C regulatory genes was examined, and varying degrees of mutations in all m5C regulatory genes were revealed. DNMT3B had the highest mutational frequency. And locations of copy number variations (CNVs) in m5C regulatory genes were displayed (Supplementary Fig. 1). The aforementioned analysis indicated significant differences in the expression levels of m5C regulatory genes between HNSCC samples and normal samples, suggesting that the differential m5C modification patterns could play a critical role in the occurrence and development of HNSCC.

**Table 1:** Clinical characteristics of patients included.

Characteristics	No. of patients (n = 20)
Gender	
Male	20 (100%)
Age (years)	
≤ 60	5 (25%)
> 60	15 (75%)
Primary site	
Larynx	18 (90%)
Hypopharynx	2 (10%)
T stage	
2	6 (30%)
3	10 (50%)
4	4 (20%)
N stage	
0	10 (50%)
1	4 (20%)
2	6 (30%)
M stage	
0	20 (100%)
Clinical staging	
II	3 (15%)
III	10 (50%)
IV	7 (35%)
Grading	
G1	2 (10%)
G2	16 (80%)
G3	2 (10%)

### Distinct m5C methylation modification patterns mediated by m5C regulatory genes

**Figure 2:**
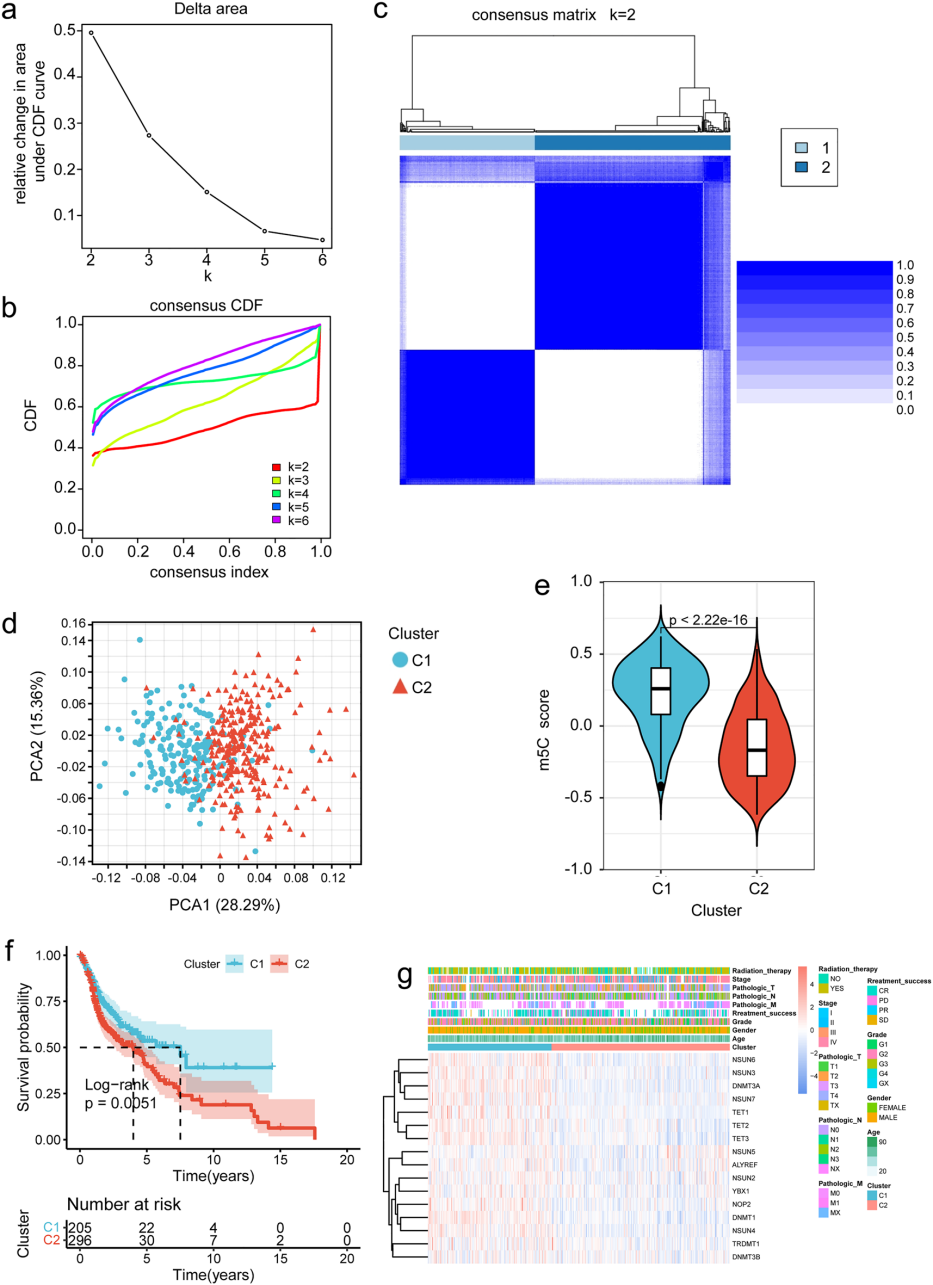
Characterization of m5C methylation patterns in HNSCC. (**a**) Relative changes in the cumulative distribution function (CDF) area for k = 2–6; (**b**) Consensus clustering cumulative distribution function (CDF) for k = 2–6; (**c**) Consensus clustering matrix for k = 2, and the color legend for consistency is on the right, indicating that the higher the consistency of the data, the bluer the color; (**d**) Principal component analysis (PCA) used to distinguish two different m5C methylation modification patterns; (**e**) GSVA demonstrated differential m5C scores between the two m5C methylation clusters; (**f**) Kaplan–Meier curves of the two clusters. **g**: Expression levels of m5C regulatory genes in the two clusters. The heatmap was constructed via the R package ggplot2.

Using the gene expression data of m5C regulatory genes, unsupervised cluster analysis was performed on TCGA tumor samples, revealing distinct classification patterns indicated by an upward trend in cumulative distribution function (CDF) values relative to the consensus index. By analyzing the CDF curve and Delta area, it was determined that the maximum difference between clusters could be obtained at the point of k = 2 when increasing the clustering index from 2 to 6 (Fig. 2a,b). Consequently, two m5C clusters, namely C1 and C2, were identified, consisting of 205 and 296 HNSCC samples, respectively. Data consistency within the same cluster was highest when K = 2 as compared to K = 3,4,5,6. (Fig. 2c, Supplementary Fig. 2). The stability of the clustering results was further confirmed using principal component analysis (PCA) (Fig. 2d).

To validate the clustering results, the m5C scores were compared between the two sets of m5C clusters using gene set variation analysis (GSVA). Significantly different m5C scores were found between the clusters, with C1 exhibiting significantly higher scores than C2 (Fig. 2e). Subsequently, the survival time and correlation with clinical factors were evaluated for each m5C cluster, demonstrating a distinct prognostic performance, while C2 exhibited a worse prognostic performance (Fig. 2f,g).

### Different m5C modification patterns were associated with different EMT levels

**Figure 3:**
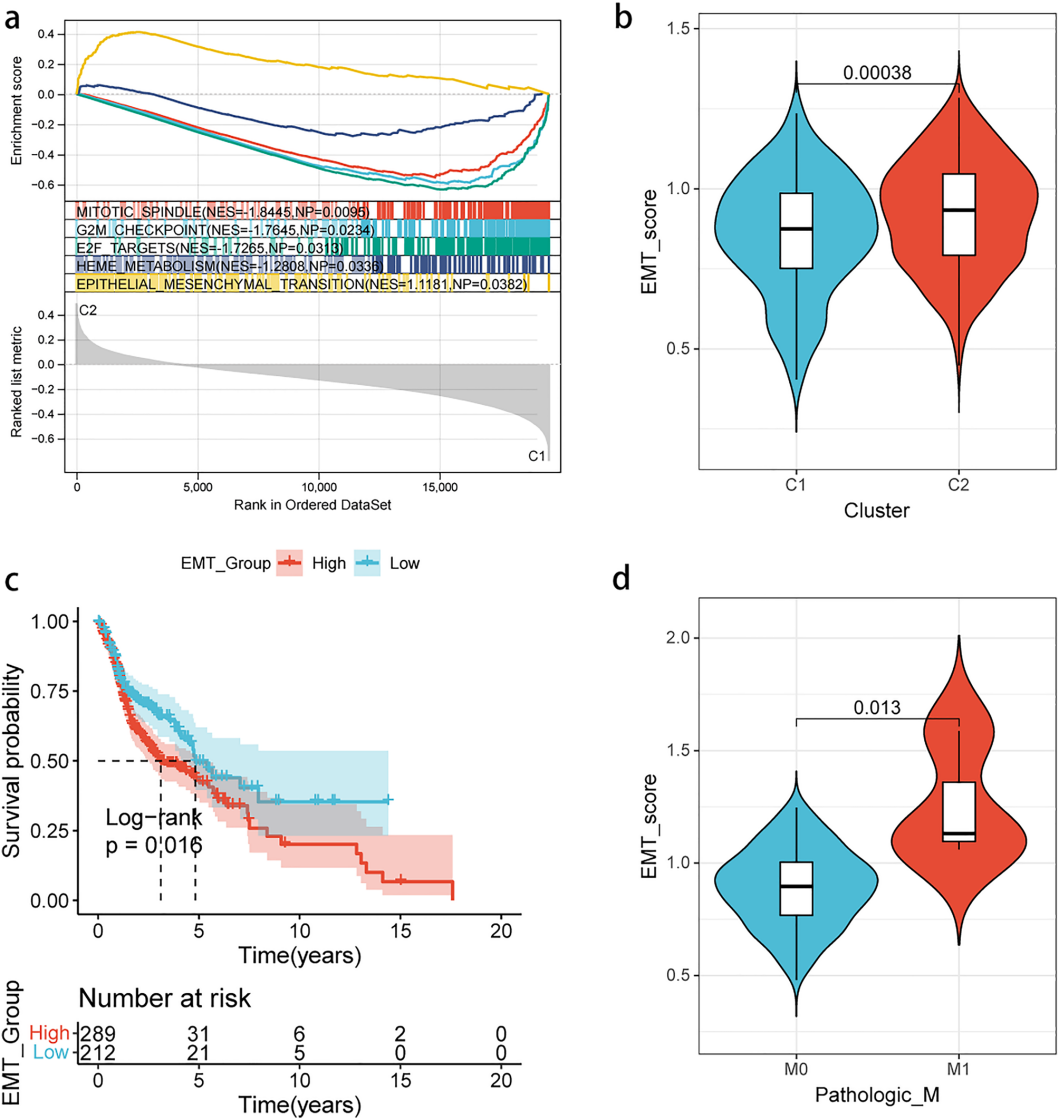
EMT status in different m5C methylation patterns. (**a**) GSEA of the two m5C clusters. EMT is enriched in C2; (**b**) EMT scores is higher in C2 based on ssGSVA; (**c**) Kaplan–Meier curves of different EMT statuses; (**d**) M stage demonstrated different EMT scores.

In order to further explore the differences in biological function between the two groups of m5C clusters, GSEA was utilized to analyze the pathways exhibiting differential enrichment. The results revealed that pathways, such as mitotic spindle, G2M checkpoint, E2F targets, and heme metabolism were enriched in C1, while EMT-associated genes were enriched in C2 (Fig. 3a). Furthermore, the differential scoring of the EMT pathway between the two groups of m5C clusters was validated by ssGSVA (Fig. 3b). Therefore, it could be speculated that m5C methylation modifications could mediate the EMT process in HNSCC samples.

Additionally, the impact of EMT levels on the prognosis of HNSCC patients was analyzed, and it was found that patients with higher EMT levels had worse prognosis, thereby explaining the poorer prognosis of C2 patients (Fig. 3c). Moreover, the relationship between EMT levels and clinical characteristics of HNSCC patients was examined, and it was noted that patients with distant metastasis exhibited higher EMT levels, while no significant differences were found in EMT levels among different grades, stages, T stages, and N stages (Fig. 3d, Supplementary Fig. 3).

### Identification of core DEGs based on m5C methylation and EMT status

**Figure 4:**
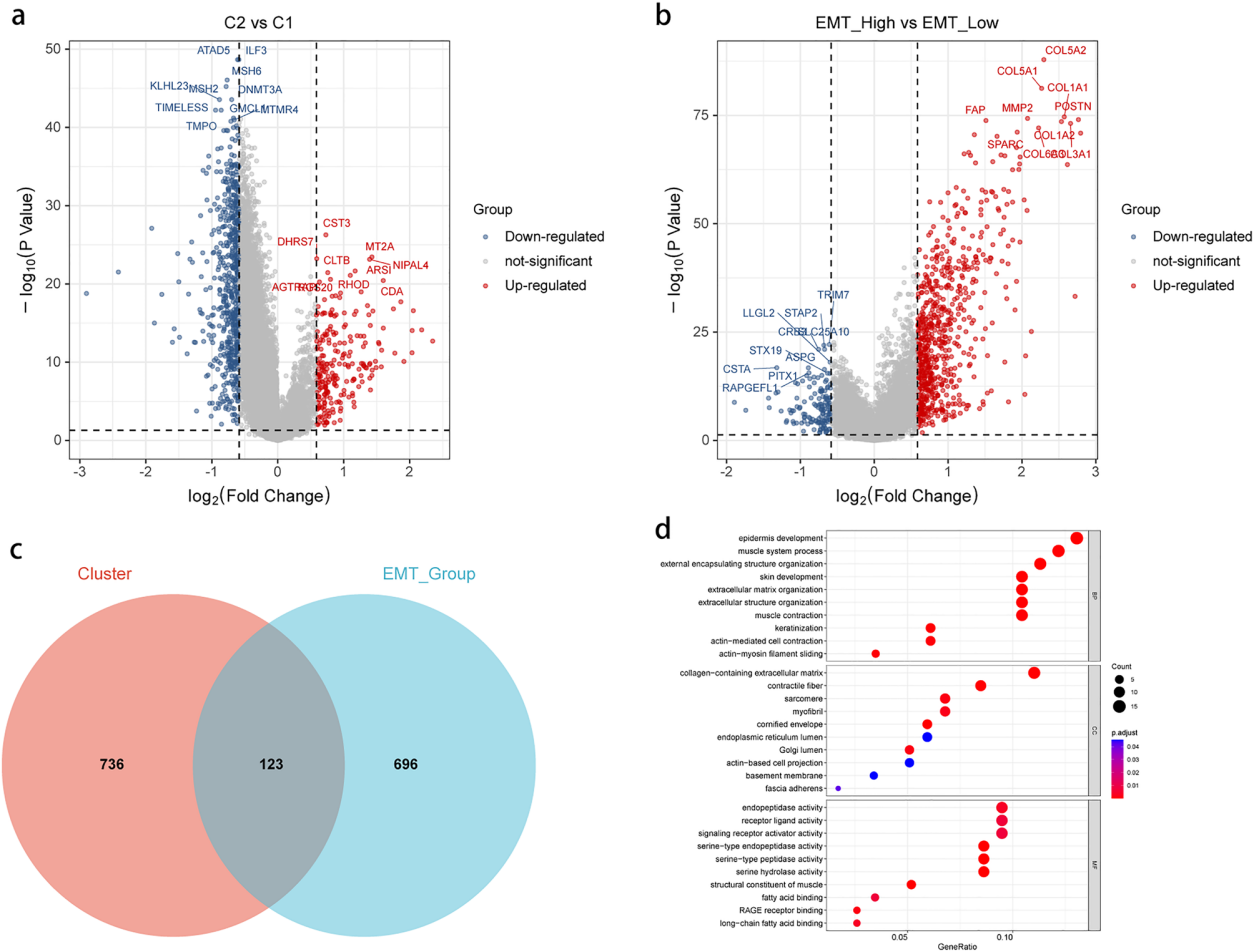
Differential gene expression analysis based on m5C methylation patterns and EMT status in HNSCC. (**a)** Volcano map of DEGs in different m5C clusters; (**b**) Volcano map of DEGs in different EMT statuses; (**c**) Venn diagram depicting 123 core DEGs in different m5C clusters and EMT groups; (**d**) GO enrichment analysis displaying the biological functions of core DEGs.

In order to further investigate the biological processes underlying the different m5C modification patterns and EMT levels, differential gene expression analysis between the m5C C2 and C1 groups was performed, and a total of 859 m5C-DEGs with significantly different distribution were screened (Fig. 4a). Differential analysis of gene expression levels between the high and low EMT score groups was then carried out, and a total of 819 EMT-DEGs with significantly different expression levels were screened (Fig. 4b). The results of the analysis about both m5C-DEGs and EMT-DEGs are presented in the “Supplementary Table 2,3”. To further analyze the inner connection between m5C modification patterns and EMT levels, we searched for intersecting genes in m5C-DEGs and EMT-DEGs. Totally, 123 overlapping genes were identified in both parts (Fig. 4c). Subsequently, in order to examine the biological functions involved in these 123 core DEGs, functional annotation of the core DEGs was performed using Gene Ontology (GO) analysis. The results of GO analysis revealed that the core DEGs were significantly enriched in biological processes (BP), such as "epidermis development", "muscle system process," and "external encapsulating structure organization." In terms of cellular components (CC), enrichment was noted in "collagen-containing extracellular matrix" and "contractile fiber." Furthermore, in molecular function (MF), enrichment was found in "endopeptidase activity", "receptor ligand activity", and "signaling receptor activator activity" (Fig. 4d). The findings from the GO enrichment analysis underscored the strong relevance of these core DEGs with the EMT process in HNSCC samples.

### Development of the prognostic model based on core DEGs

**Figure 5:**
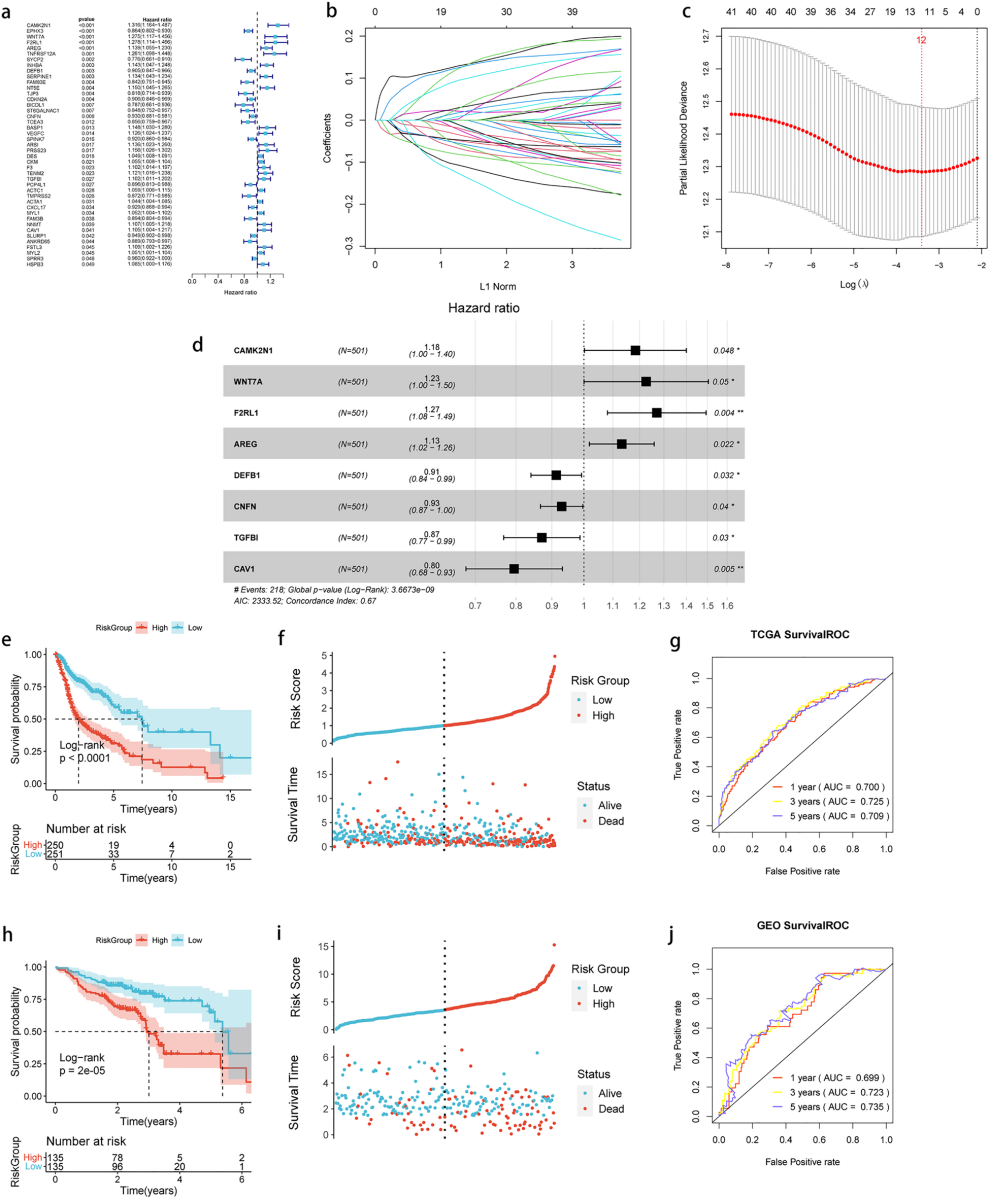
Development and validation of the prognostic model based on m5C cluster and EMT in HNSCC. (**a**) Forest plot of univariate Cox regression analysis for 123 core DEGs; (**b**, **c**) LASSO regression analysis with minimal lambda value; (**d**) Forest plot of multivariate Cox regression analysis for 8 genes; **e**: Kaplan–Meier curves of high and low-risk groups in the training dataset; (**f**) Distribution of risk score and survival status of HNSCC patients with different risk scores in the training dataset; (**g**) Time-dependent ROC curve analysis in the training dataset; (**h**) Kaplan–Meier curves of high- and low-risk groups in the validation dataset; (**i**) Distribution of risk score and survival status of HNSCC patients with different risk scores in the validation dataset; (**j**) Time-dependent ROC curve analysis in the validation dataset.

Using the 123 core DEGs, univariate Cox regression analysis was performed and a total of 43 genes with significant prognostic correlation were screened at a threshold of *P* < 0.05 (Fig. 5a). Subsequently, LASSO regression analysis was conducted on 43 genes to remove genes that were highly similar to others, and 12 genes were finally screened (Fig. 5b,c). Stepwise COX regression analysis was used to identify optimal gene combinations, and 8 model genes were finally identified (Fig. 5d). The Riskscore model was developed based on the regression coefficients of the 8 model genes and their expression levels in TCGA training dataset. The Riskscore model was formulated as follows:

$$\begin{aligned} Risk & = 0.168840761*CAMK2N1 + 0.204194584*WNT7A + 0.239159145*F2RL1 \\ & \quad + 0.123999752*AREG - 0.090946852*DEFB1 \\ & \quad - 0.073033023*CNFN - 0.138134256*TGFBI* - 0.229116348*CAV1 \\ \end{aligned}$$

The Riskscore of each sample was computed, and the samples in TCGA training dataset and GEO validation dataset were divided into high and low groups according to the median of the Riskscore, respectively. The results of the Kaplan–Meier analysis demonstrated that patients with high-risk scores had significantly shorter overall survival (OS) compared with those in the low-risk group (Fig. 5e,f), which was consistent with the findings observed in the GEO validation set (Fig. 5h,i). To validate the prognostic model, receiver operating characteristic (ROC) curve analysis was performed to evaluate its accuracy. The results from TCGA training dataset showed that the area under the ROC curve (AUC) values for 1-, 3-, and 5-year survival were 0.7, 0.725, and 0.709, respectively (Fig. 5g). Similarly, the AUC values for the ROC curves of the GEO validation dataset at 1-, 3-, and 5-year survival were 0.699, 0.723, and 0.735, respectively (Fig. 5j). These findings indicated that the 8-gene signature could serve as an effective predictive indicator for clinical outcomes in HNSCC samples.

### Independent evaluation of the prognostic model and nomogram development

**Figure 6:**
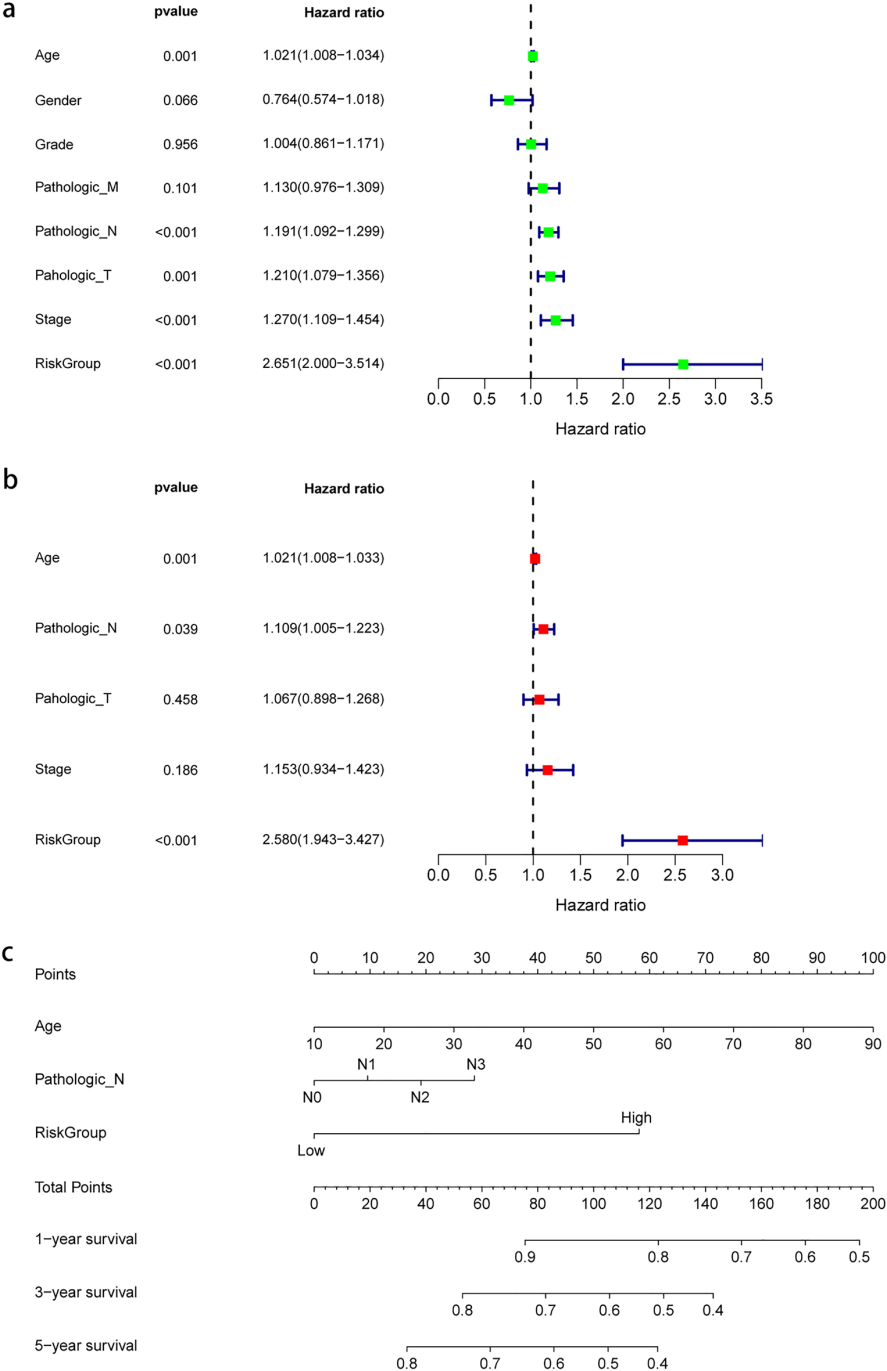
Independent validation of the risk model and development of the nomogram. (**a**) Univariate Cox regression of the 8-gene risk model and clinical data; (**b**) Multivariate Cox regression of the 8-gene risk model and clinical data; (**c**) Nomogram developed based on the results of the multivariate Cox regression analysis.

Univariate Cox regression and multivariate Cox regression analyses were carried out to independently evaluate the prognostic significance of risk group and clinical features. The results of the univariate Cox regression analysis showed that age, N stage, T stage, stage, and risk group had significant prognostic implications in HNSCC samples (Fig. 6a). Furthermore, the multivariate regression analysis revealed that only age, N stage, and risk group remained significantly associated with prognosis (Fig. 6b). It is interesting that the risk models we constructed in either univariate Cox regression or multivariate Cox regression are significantly high in Hazard ratio. Additionally, a nomogram was developed to assess the prognosis of HNSCC patients (Fig. 6c). These findings suggested that the 8-gene signature could serve as a valuable prognostic tool for evaluating HNSCC patients.

### Expression pattern analysis of the gene signature

**Figure 7:**
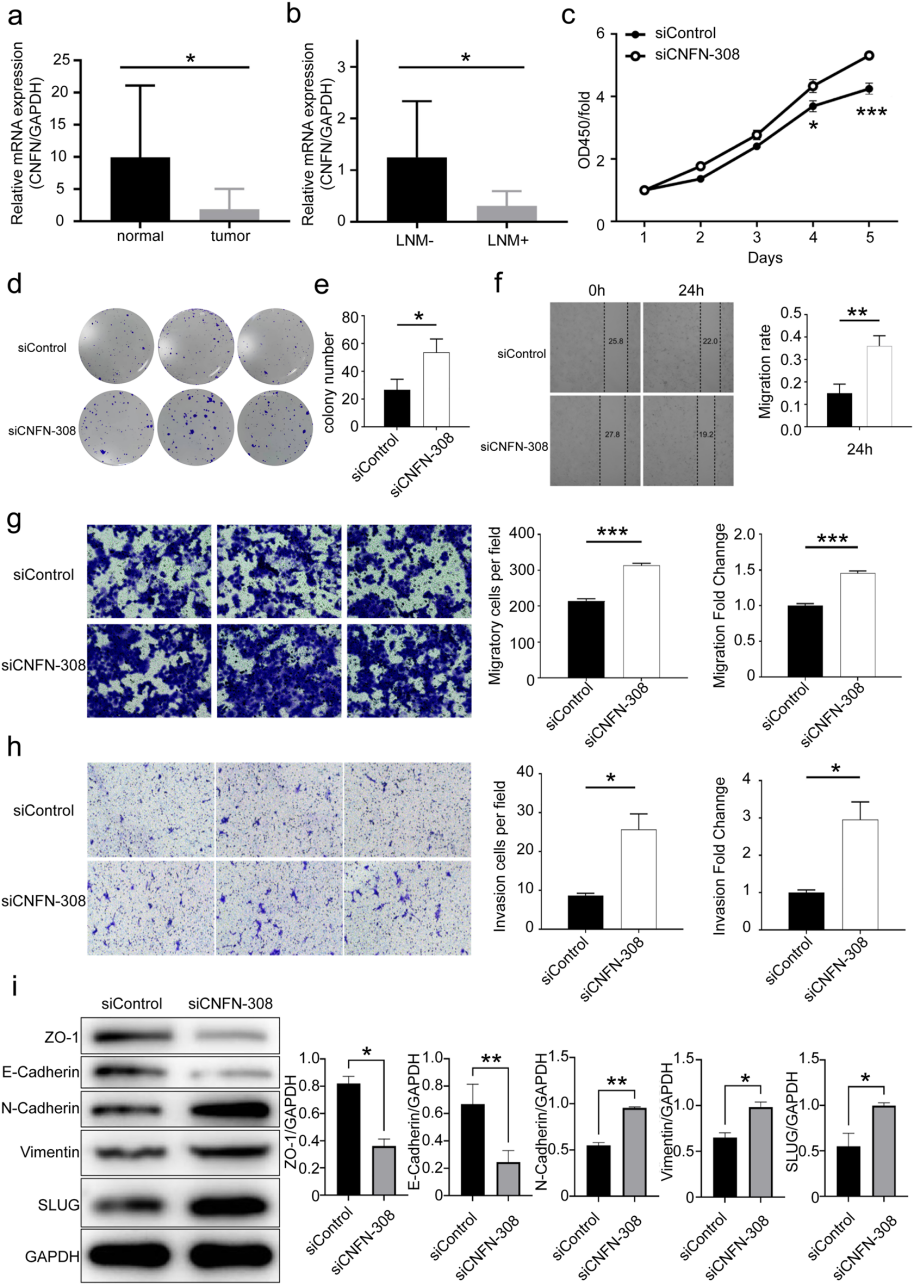
Biological functions and mechanistic studies of the prognostic biomarker. (**a**) Expression level of CNFN in tumor and normal tissues detected using RT-qPCR; (**b**) Expression level of CNFN in tumor tissues between lymph node metastasis and non-lymph node metastasis detected using RT-qPCR; (**c**–**e**) Cell proliferation capacity was validated using colony formation and CCK-8 assays; **f**: Cell migration ability was evaluated using wound healing assay; (**g**) Transwell migration assay was used to assess the effects of CNFN on cell migration ability; (**h**) Transwell invasion assay was utilized to evaluate the effects of CNFN on cell invasion ability; (**i**) Western blotting was used to detect changes in the expression levels of EMT-associated markers after silencing of CNFN.

To gain further insights into the functional implications of the 8-gene signature in HNSCC, the role of CNFN was specifically investigated. It was found that CNFN exhibited significantly higher expression levels in normal tissues compared with HNSCC tissues. There was a trend toward longer OS and disease-free survival (DFS) in patients with high levels of CNFN expression compared to those with low levels of CNFN expression, although this was not statistically significant (Supplementary Fig. 4). The results of RT-qPCR from clinical samples also confirmed the downregulation of CNFN in HNSCC tissues (Fig. 7a). Notably, after comparing the expression levels of CNFN between patients with lymph node metastasis (LNM +) and those without lymph node metastasis (LNM-), it was indicated that LNM- patients had significantly higher CNFN expression levels (Fig. 7b). These findings suggested that CNFN could serve as a potential prognostic indicator and a marker for LNM in HNSCC patients.

In additional, we analyzed the expression of each m5C regulator in LNM + vs LNM-. However, only some of the m5C regulators were differentially expressed. Among the differentially expressed genes present, DNMT1, NSUN2, NSUN4, and TET2 were highly expressed in LNM + tumor tissues while NSUN5 and NSUN7 expression was reduced (Supplementary Fig. 5).

### Functional and mechanistic assessment of CNFN in HNSCC

Further investigation into the functional role of CNFN in HNSCC is deemed necessary. The HNSCC lymph node metastasis cell line UM-SCC-17B was utilized for subsequent studies. Clonogenic assay revealed a significant increase in the number of colonies formed upon transfection with CNFN siRNA (Fig. 7c,d). Additionally, CCK-8 assay demonstrated a significant difference in cell proliferation ability following transfection with CNFN siRNA (Fig. 7e). These findings indicated that CNFN has the capability to suppress cell proliferation in HNSCC. The results of wound healing assay exhibited a more pronounced wound healing ability in UM-SCC-17B cells upon silencing of CNFN (Fig. 7f). Furthermore, the results of Transwell migration and invasion assays also indicated an increase in cell migration and invasion capabilities upon silencing of CNFN (Fig. 7g,h). These findings might explain why CNFN expression level was lower in patients with LNM.

Finally, the involvement of CNFN in regulating the metastatic potential of HNSCC through the EMT pathway was explored. After transfection with CNFN siRNA, protein of UM-SCC-17B were extracted, and the expression levels of EMT-related proteins were examined. The results showed a decrease in expression levels of ZO-1 and E-Cadherin upon silencing of CNFN, while the expression levels of N-Cadherin, Vimentin, and SLUG were elevated (Fig. 7i). This suggested that CNFN could regulate HNSCC metastasis by influencing the EMT pathway.

## Discussion

HNSCC is widely acknowledged as a complex and heterogeneous disease. However, the absence of reliable early screening methods poses significant challenges to the early diagnosis and treatment of HNSCC^33^. Recent research indicated that tumor initiation and progression are influenced not only by genetic mutations, but also by alterations in the epigenetic profile^34^. DNA methylation, histone modifications, and RNA methylation have been found to play important roles in cancer^35–37^. The significance of RNA methylation modifications in cancer is gaining significant recognition. Among these modifications, m5C methylation, which is a prevalent form of RNA methylation present in most RNAs, has been implicated in the progression of several malignancies, including hepatocellular carcinoma (HCC), breast cancer, gastric cancer and HNSCC^14,27,38,39^. A previous study indicated that m5C regulatory genes can serve as prognostic markers for HNSCC^40^. Additionally, another study identified a 3-gene signature consisting of NSUN5, DNMT1, and DNMT3A, acting as independent prognostic factors for HNSCC^28^. Furthermore, recent research suggested a close association between m5C methylation of mitochondrial tRNA and metastasis in HNSCC, significantly contributing to understanding the role of m5C methylation modifications in HNSCC^14^. However, the regulation and biological mechanisms of RNA m5C methylation in tumors have still largely remained elusive and require further investigation.

The present research identified two distinct m5C modification patterns in HNSCC patients that exhibited significant prognostic differences. Furthermore, it was found that these two m5C modification patterns were associated with different levels of metastasis and EMT. It is broadly accepted that EMT plays a crucial role in tumor metastasis^41^. Studies suggested that the epigenetic landscape plays a significant role in controlling EMT plasticity. A series of histone modifications have been found to regulate the EMT process, and DNA methylation has also been found to regulate the EMT process^42–45^. Moreover, RNA methylation, especially RNA m6A methylation, has been extensively reported to play a role in regulating EMT. Previous research indicated that the loss of the m6A methylation eraser METTL3 could lead to tumor metastasis and EMT^46^. Additionally, another m6A methylation eraser, FTO, has also been implicated in the regulation of EMT levels^47^. To date, there is a lack of specific studies investigating the role of m5C methylation modifications in the regulation of the EMT process. Thus, this research aimed to fill this knowledge gap and provide novel insights for future investigations on the metastatic process and the regulatory mechanisms of EMT in cancer.

One of the key findings in this study is the development of an 8-gene signature that combines m5C methylation patterns and tumor EMT profiles to predict prognosis in HNSCC. This risk score model has demonstrated its efficacy in reliably predicting OS outcomes in HNSCC patients, and it could serve as an independent prognostic factor. The eight genes (CAMK2N1, WNT7A, F2RL1, AREG, DEFB1, CNFN, TGFBI, and CAV1) included in the signature have been extensively studied and are known to be involved in tumor progression and EMT process. For instance, CAMK2N1, which is upregulated in locally invasive and metastatic prostate cancer, has been identified as an EMT-related gene with implications in cancer progression^48^. WNT7A is a member of the Wnt signaling pathway, which plays a crucial role in various biological processes, and it is closely associated with tumor initiation and progression^49^. Amphiregulin (AREG) is a member of the epidermal growth factor (EGF) family. Studies have also demonstrated that AREG could participate in the cancer progression mediated by EGFR^50,51^. The relationship between TGF-β1 and EMT has been clearly established^52^. Similarly, CAV1, a well-established tumor suppressor gene, has garnered significant recognition for its influential role in various diseases^53^. For the remaining genes, although some reports have explored their functions in tumors, the existing research is not comprehensive enough. Further in-depth studies are warranted to unravel the mechanisms underlying the progression and metastasis of HNSCC.

CNFN, also known as cornifelin, is prominently expressed in the esophagus and skin and was initially discovered during psoriasis research^54^. While CNFN's precise function remains unclear, limited studies suggest its significance in maintaining normal skin and mucosal function^55^. In recent bioinformatics studies concentrating on HNSCC, CNFN has emerged as an independent prognostic factor, with higher expression levels associated with improved prognosis^56^. Additionally, CNFN may be associated with LNM in HNSCC^57^. However, the involvement of CNFN in HNSCC progression and its specific molecular mechanisms have not yet been explored. The present study found a decrease in CNFN expression in tumor tissues of HNSCC patients with LNM, providing the first evidence of CNFN as a tumor suppressor gene, inhibiting the proliferation, migration, and invasion abilities of HNSCC cell lines. Additionally, we investigated the potential mechanisms through which CNFN could influence tumor-associated LNM. The experimental findings suggested that CNFN could impact the EMT process in HNSCC cell lines. This study presents conclusive evidence regarding the functional role of CNFN in HNSCC, providing new insights for predicting HNSCC patients’ prognosis and LNM.

However, it is important to acknowledge the limitations of the present study. Firstly, this study only speculates on the modification pattern of m5C in HNSCC based on the expression of m5C regulators. To examine the RNA m5C methylation modification, BS-RNA sequencing is required. We hope to utilize this technology in future studies to enable the clarification the RNA m5C modification in HNSCC. Secondly, based on our current study, we cannot make conclusions about the causal relationship between methylation and EMT. It can be speculated that different methylation levels in head and neck squamous carcinomas may have different EMTs, as we described. Further experiments need to be carried out to clarify the regulatory relationship between m5C and EMT by interfering with the expression of m5C regulators and detecting m5C modification patterns.

Meanwhile, the experimental part of this study still needs to be discussed. Only 20 patients with HNSCC were included in this study, and the anatomical sites of the tumors were only laryngeal and hypopharyngeal carcinoma. In the RT-qPCR assays we performed on clinical samples from 20 patients, the expression of each m5C regulator was not entirely consistent with what we analyzed in the TCGA database. This specificity of the anatomical site may have contributed to the bias of our results. It is also possible that the use of only one HNSCC cell line in our study may cause bias in our results. Meanwhile, F2RL1 and DEFB1 in signatures have not been validated by experiment. In further studies, genes in our prognostic signature must be studied in more HNSCC cell lines.

## Conclusions

In summary, distinct m5C methylation patterns were revealed in HNSCC patients. Differential levels of EMT were noted among patients with different m5C methylation features. Furthermore, an 8-gene signature with strong predictive value for patient prognosis was identified. Additionally, the functional role of one of the gene signatures, CNFN, in HNSCC, along with its potential mechanisms underlying tumor-associated LNM, was elucidated. The intricate interplay among m5C methylation, gene signatures, and HNSCC biology was unveiled, contributing to a deeper understanding of the complex nature of this disease and opening new avenues for future research and clinical applications.

### Electronic supplementary material

Below is the link to the electronic supplementary material.

Supplementary Table 1.

Supplementary Table 2.

Supplementary Table 3.

Supplementary Figures.

## Data Availability

Publicly available data sets can be found in https://portal.gdc.cancer.gov/ and GEO DataSets (GSE65858, https://www.ncbi.nlm.nih.gov/). The data that support the findings of this study are available on request from the corresponding author.

## Abbreviations

m5C: 5-Methylcytosine
EMT: Epithelial-mesenchymal transition
HNSCC: Head and neck squamous cell carcinoma
TCGA: The Cancer Genome Atlas
DEGs: Differentially expressed genes
FPKM: Fragments per kilobase per million mapped reads
GSEA: Gene Set Enrichment Analysis
ssGSEA: Single-sample gene set enrichment analysis
GSVA: Gene set variation analysis
NES: Normalized enrichment score
PCA: Principal component analysis
GO: Gene Ontology
BP: Biological processes
CC: Cellular components
MF: Molecular function
OS: Overall survival
DFS: Disease-free survival
LNM: Lymph node metastasis
LNM: Lymph node metastasis
