# Supplementary material for: Identification and validation of novel prognostic signatures based on m5C methylation patterns and tumor EMT profiles in head and neck squamous cell carcinoma
Source: Sci Rep. 2023 Oct 31;13:18763. doi: 10.1038/s41598-023-45976-6 (PMC10618291; doi:10.1038/s41598-023-45976-6)

Supplementary Information

Identification and Validation of Novel Prognostic Signatures Based on m5C Methylation Patterns and Tumor EMT Profiles in Head and Neck Squamous Cell Carcinoma

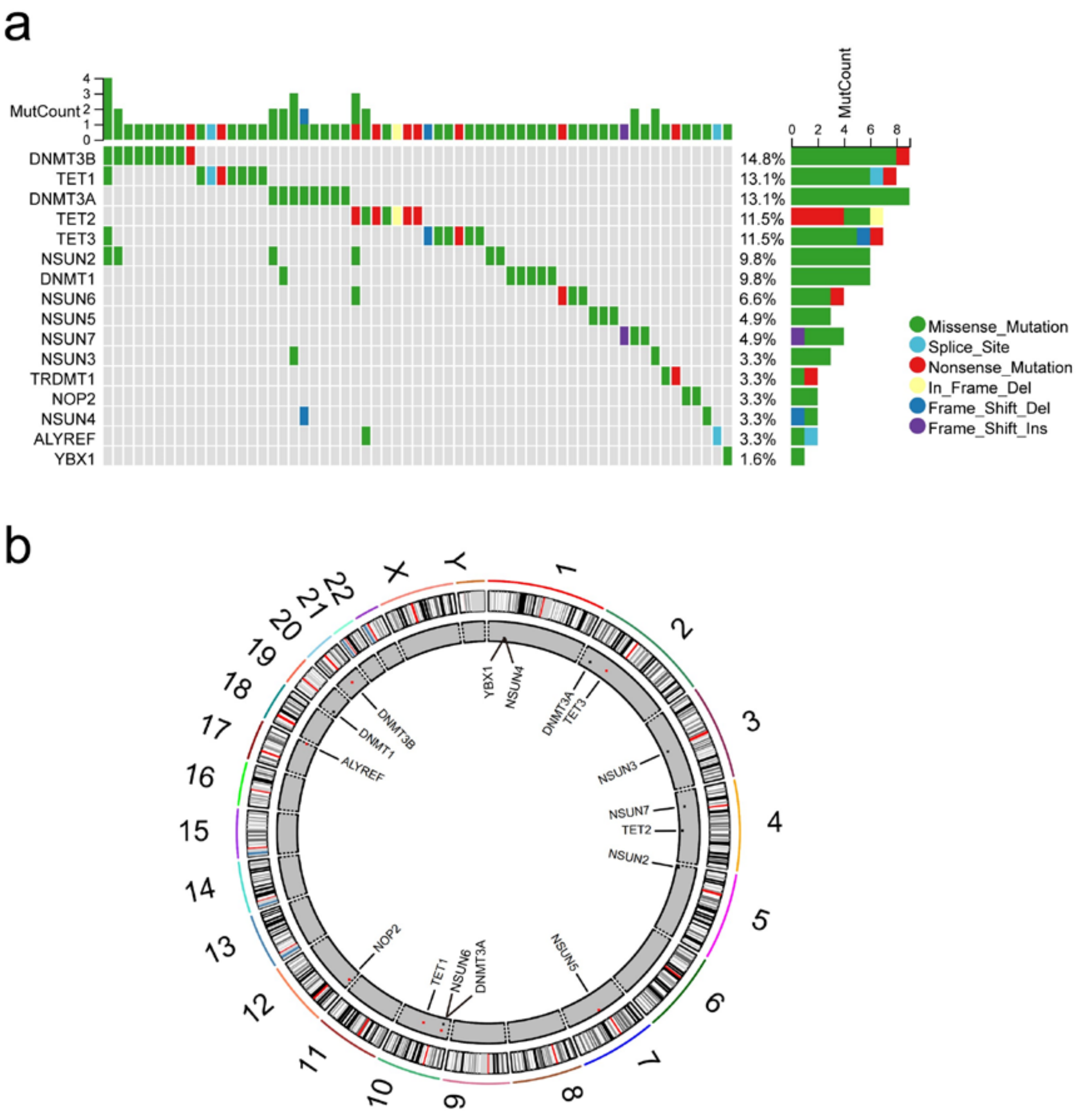

Supplementary Figure 1. Mutation mapping of m5C regulatory genes in HNSCC cohort. **a**: Mutational status of the m5C regulatory genes; **b**: CNV profile of the m5C regulatory genes on chromosomes

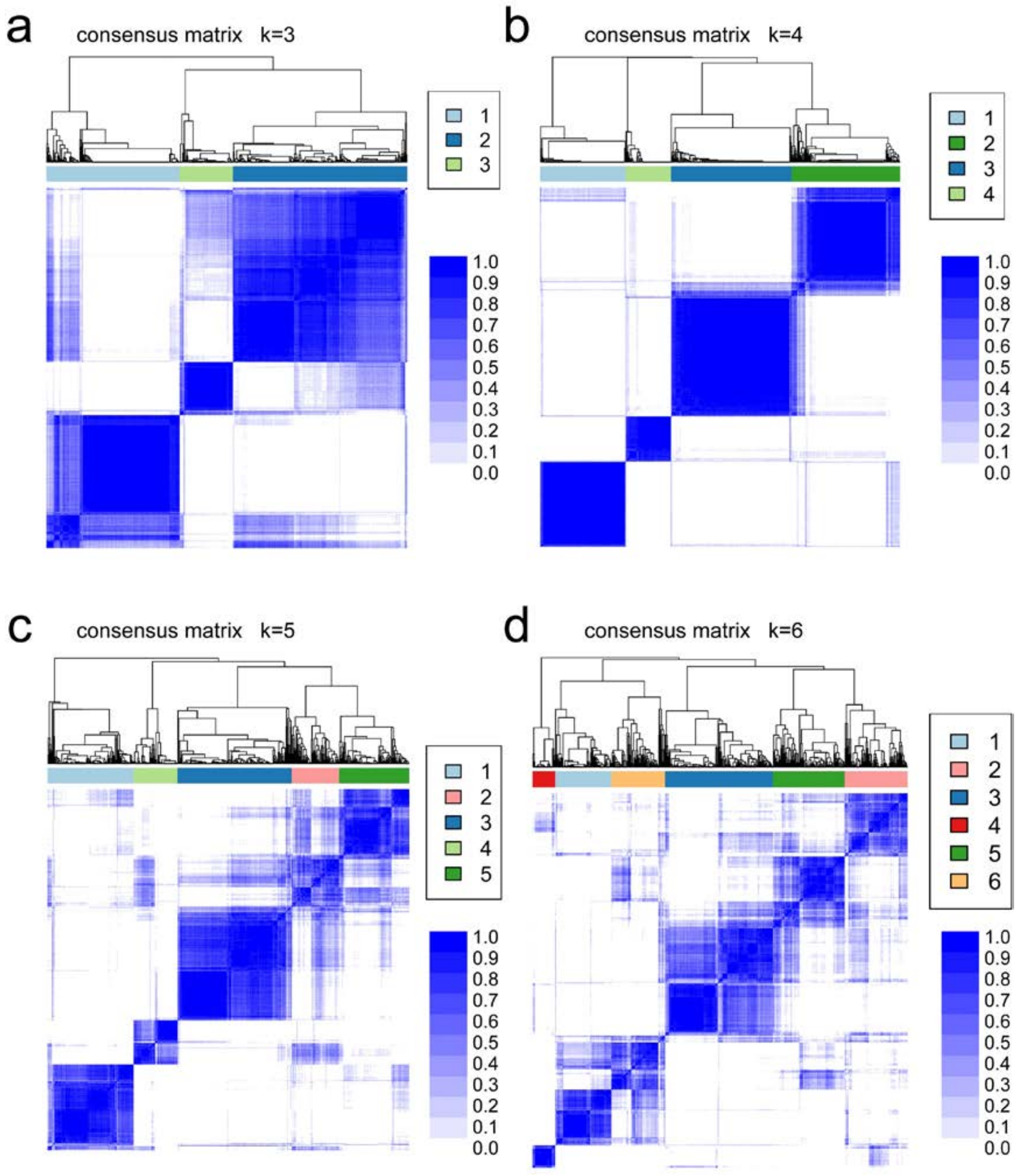

Supplementary Figure 2. Consensus clustering matrix for k = 3, 4, 5, 6.

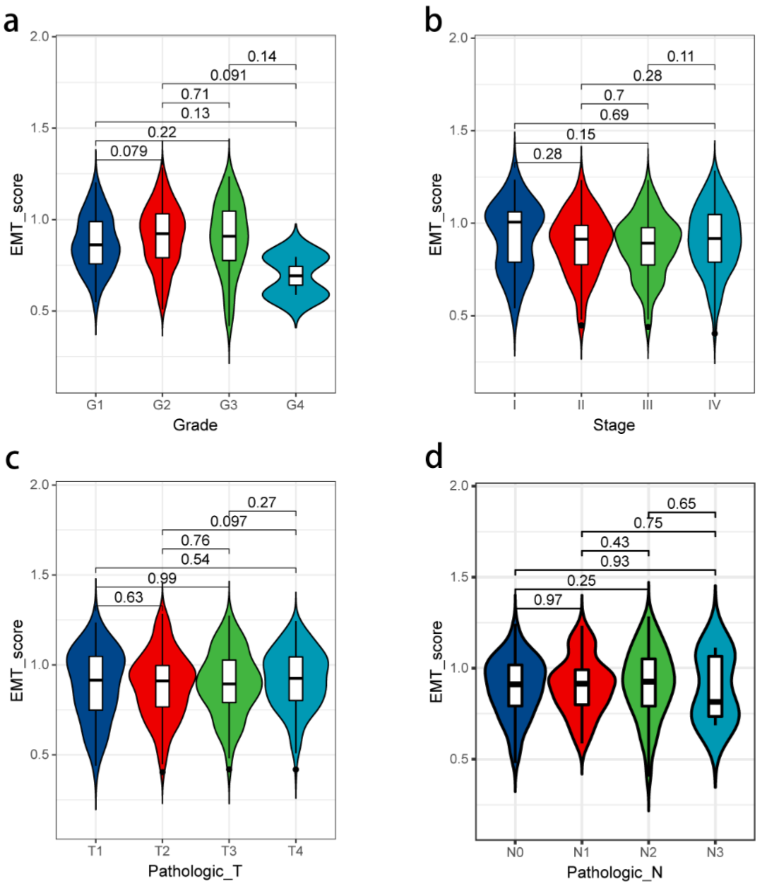

Supplementary Figure 3. EMT score of different grades, stages, T stages, and N stages.

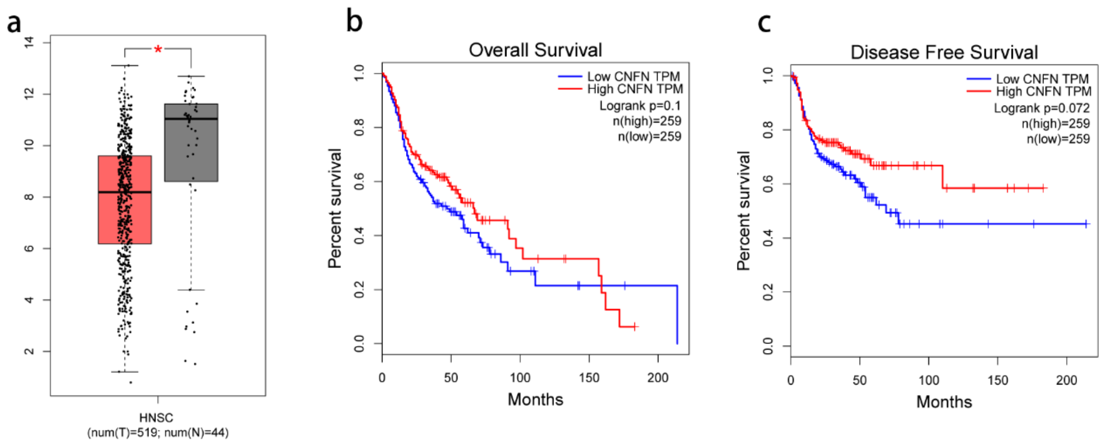

Supplementary Figure 4. expression levels of CNFN in HNSCC. a. expression levels of CNFN in normal tissues compared with HNSCC tissues; b and c. OS and DFS of different CNFN expression levels.

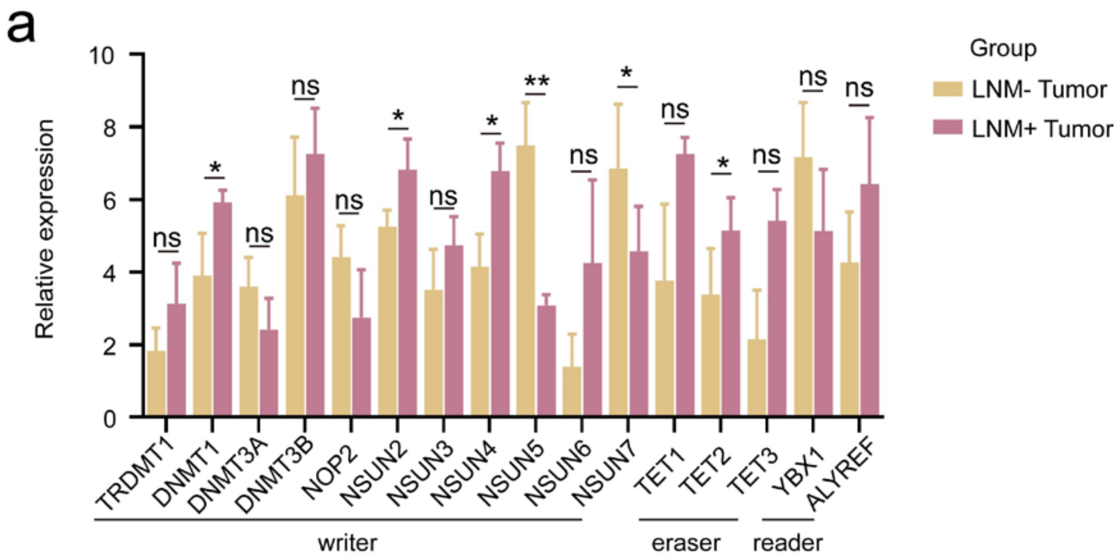

Supplementary Figure 5. Expression levels of 16 m5C regulators in clinical samples about LNM+ vs LNM-.

Uncropped western blots

ZO-1

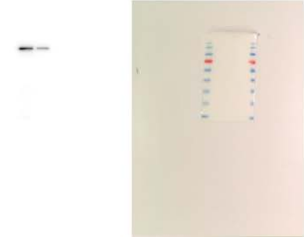

SLUG

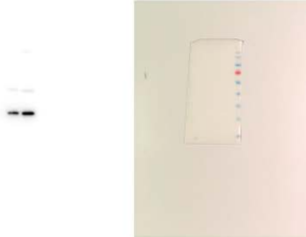

E-Cadherin

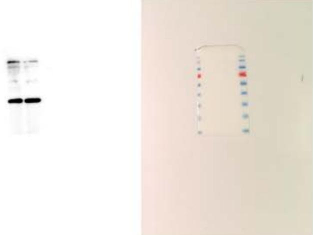

Vimentin

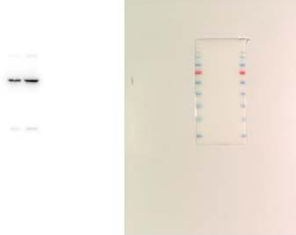

N-Cadherin

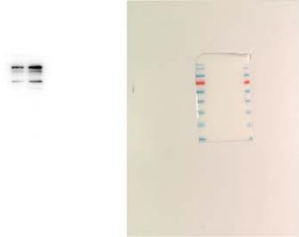

GAPDH

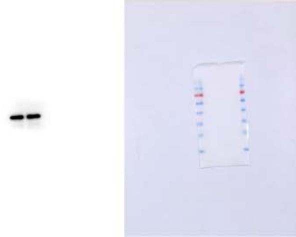

Supplement: Supplementary file 4 — Supplementary Figures. [file 41598_2023_45976_MOESM4_ESM.pdf]
